# Editorial Notes

**Published:** 1929

**Authors:** 


### Editorial Notes

**Installation of Chancellor, University of Bristol.**

THE installation of the Right Hon. Winston Spencer Churchill, P.C., C.H., as Chancellor of the University of Bristol will take place on Friday, 13th December, and it is hoped that he will address the undergraduates on the morning of Saturday, 14th December. The new Chancellor will also formally open Wills Hall, the new residential college for men.

**Wills Hall.**

WILLS HALL, which is situated on Durdham Down, at the top of Parry's Lane, has accommodation for 140 students. The full number is now in residence, with Mr. H. Norton Matthews, M.A., in charge as Warden. Mr. Norton Matthews's record as a Master at Clifton College and his many-sided interests augur well for the prosperity and contentment of Wills Hall. When the Charter was first granted in 1909 few of us contemplated the way in which Bristol would develop into a residential University. Then it seemed as though we should be content with a scattered tribe of undergraduates, some lucky in having Bristol homes, others drably facing their studies from such lodgings as their slender purses might secure. Now our horizon has been widened by great halls of residence such as were formerly attainable in Oxford and Cambridge alone.

**Bristol Medical School.**

WHILST we congratulate ourselves on our progress, and record with satisfaction every new development, it is good to look back at our past occasionally. There will be an opportunity for such a glance backward in a few years, and it is as well to be reminded in time that the year 1933 is the centenary of the foundation of the Bristol Medical School. The *Bristol Gazette* for October 24th, 1833, records its foundation thus: "The Bristol Medical School, Monday, October 14th, 1833. This Institution was opened this morning by Dr. Carrick, who delivered the preliminary address." Dr. Carrick was physician at the Infirmary from 1810 to 1834, and was a man of great ability. He played a considerable part in bringing rival schools of anatomy and surgery into harmonious unity. He also persuaded his Infirmary colleagues to invite the co-operation of the medical staff of the newly-founded General Hospital in forming the Medical School. We may well believe that this task needed all his tact and all that mildness which an Infirmary patient enshrined in doggerel verse :—

"Dr. Carrick he comes in
So meek, so mild as anything,
Saying, 'How are you to-day, my child ?
Your pains with patience you must bear,
And we will seek for your cure.'"

Carrick had had other distinctions in the same year, for he presided at the first annual meeting of the Provincial Medical and Surgical Association which was held in Bristol in 1833, and this meeting is reckoned as the first annual meeting of the British Medical

Association. So that there are two centenaries due in 1833, that of the Bristol Medical School and that of the first British Medical Association meeting in Bristol.

We might begin to dream and plan again for our successors, and we would dream perhaps of a second Carrick breaking the barriers between the Infirmary and the Hospital for good and all.

**Annual Dinner of the Society.**

The first annual dinner of the Bristol Medico-Chirurgical Society was held at the Berkeley Café, Clifton, on Friday, 18th October, Mr. Ferrier Walters, the President, being in the Chair. Forty members and guests attended and spent a most enjoyable evening. Professor Berry, who has recently vacated the Chair of Anatomy in the University of Melbourne, and come to live in Bristol, proposed the toast of the "Society" in a witty speech, full of interesting information about medical education in Australasia, Canada and the United States. Dr. Ormerod, the immediate Past-President of the Society, in the course of the evening mentioned that not only his father but his grandfather and great-grandfather had practised medicine in Bristol (and most of the audience were aware that Dr. Ormerod has a son, either just qualified or sitting for his final examinations). This dinner, which the Medico-Chirurgical Society has, after fifty-five years' existence, at last established, seems to fill a gap in the social organization of the profession where it was really wanted. It has started well, and promises to be the beginning of a long series of successful and hospitable gatherings.

**The Eastman Dental Clinic, London.**

MR. L. E. CLAREMONT has been appointed Director of the Eastman Dental Clinic in London, and will shortly be leaving Bristol, to the regret of all his friends and colleagues here. It is, however, an appointment on which he is heartily to be congratulated, and it reflects honour on our Dental School that he has been selected as the chief of this new and important institution.

The building has been provided by the generosity of Mr. George Eastman, an American, the present chairman of Kodak Limited, Rochester, N.Y.

Among many gifts to Rochester this philanthropist built a Dental Clinic some fifteen years ago, designed and equipped for carrying out intensive treatment for children—orthodontia playing a big part. The building contains a tonsil and adenoid department, with an operating theatre and twenty beds. The social service is highly organized and the financial status of the patients carefully considered. Each subscribes something towards the cost of treatment. Mr. Eastman considers that patients who pay something, however small, value what they are getting more than those who are treated free. The staffing of the dental side consists entirely of post-graduates acting in a similar capacity to house surgeons. The anæsthetists and throat surgeons attend on certain days from the Rochester Hospital. Apart from the clinical side, the building contains a public lecture theatre, where elementary instruction is given on dental prophylaxis to expectant and nursing mothers, and also the children.

Another activity is the School of Women Dental

Hygienists. The students receive a six months' course in elementary anatomy and physiology, dental radiology, the cleaning and scaling of teeth, and the examination of teeth and mouths generally. Squads of the qualified hygienists are attached to the clinic with a dental surgeon in charge of each squad. They work systematically through the schools, cleaning, scaling, and examining the teeth every three months. Abnormalities are reported to the dental surgeon in charge of the particular squad concerned, and the child is sent to him at the clinic for the necessary treatment.

This work has proved such a success that Mr. Eastman decided to offer a similar gift to Europe, and through the foresight of Lord Riddell the Royal Free Hospital in London has been privileged to undertake the work. The gift in English currency amounts to £200,000. Lord Riddell, the Chairman of the Royal Free Hospital, and the Treasurer, Sir Albert Levy, have given £50,000 each towards the endowment fund.

The completed building will contain seventy-four dental chairs, sixty-eight of which will be fitted with electrical equipment of the latest design. The surgical side is somewhat larger than in Rochester, provision having been made for an operating theatre and forty-five beds, some of which are intended for plastic jaw surgery. There is a larger up-to-date pathological department. Permission has been given to treat adult patients in London, and the hope has been expressed that a Dental School for women should be inaugurated after the children's clinic has been fully established. With this end in view the architectural design, apart from the public lecture theatre with a cinematograph, etc., includes a mechanical and prosthetic department, and various other lecture and demonstration rooms.

The foundation-stone was laid by His Royal Highness the Prince of Wales in April, 1929. It is hoped that the official opening of the building will take place in the summer of 1930.

**The Wellcome Museum of Medical Science.**

WE are always proud to read of the achievements of men who have studied and worked in Bristol. Those who remember Dr. S. H. Daukes as a House Physician at the Royal Infirmary in 1905 will be pleased to know of his success as Curator of the Wellcome Museum of Medical Science as described in the *British Medical Journal* of October 12th :—

"Dr. S. H. Daukes is one of those fortunate men who have lived to realize a great ambition, made possible by the munificence of Dr. Henry S. Wellcome. As a student Dr. Daukes was impressed with the dullness and deficiencies of most museums attached to medical schools, and conceived a scheme for their regeneration. He had to wait for his opportunity ; it came to him first in 1917–18, when he was placed in charge of the Army School of Hygiene at Leeds. He resolved to teach his men objectively ; trenches were cut, dug-outs made, latrines erected, incinerators set up, and kitchens built ; these became his school of army sanitation. The success of the school was noted by Dr. Andrew Balfour, who perceived that the officer in charge was a born teacher, one who was brimming over with fresh ideas of a kind which could be put into practice. Dr. Balfour invited Dr. Daukes to become Curator of the Wellcome Museum of Tropical Medicine."

This museum was started in 1914 by Dr. Andrew Balfour, who brought as its nucleus the exhibits he had collected, on behalf of the Sudan Government, for the great Hygiene Museum at Dresden in 1911, and later for the Ghent Exhibition. The aim was to establish a museum on graphic lines illustrating by pictures, charts and specimens the complete story of nearly all the tropical diseases. At first the collection was housed in Henrietta Street, Cavendish Square, with Dr. George Buchanan as Curator until 1919, when Dr. Daukes was appointed to be his successor. The Wellcome Museum is now situated at Endsleigh Court, and is no longer confined to Tropical Diseases. It deals with all branches of Medical Science, and offers to the postgraduate and advanced student a complete account of various diseases by all appeal to the eye.

It is interesting to recall that Mr. L. W. G. Malcolm of the Wellcome Medical History Museum is also connected with Bristol, where he was for some years Dr. Bolton's assistant at the Museum and Art Gallery.

